# Editorial: Decision making and its pathologies: a translational animal-to-human overview

**DOI:** 10.3389/fnbeh.2026.2021188

**Published:** 2026-10-27

**Authors:** Enrico Patrono, Ron Keiflin, Jean-Christophe Cassel

**Affiliations:** 1 New York University—Prague, Prague, Czechia; 2 Institute of Physiology, Academy of Sciences of the Czech Republic (ASCR), Prague, Czechia; 3 University of California, Santa Barbara, Santa Barbara, CA, United States; 4 Universite de Strasbourg, Strasbourg, France

**Keywords:** behavioral alterations, decision making, neurobiological modifications, psychiatric manifestations, translational approaches

At the basis of psychological conditions, whether psychiatric or neurodegenerative, exists a systemic, maladaptive choice behavior. Making decisions requires an organism to navigate uncertainty, gauge environmental flux, compute the metabolic cost of effort, and satisfy reward needs. When these executive functioning pipelines break down, the result is pathological decision-making. Rather than viewing these deviations as simple failures of will, modern clinical neuroscience conceptualizes them as distinct biological breakdowns in brain communication and valuation. A growing body of groundbreaking scientific papers has begun mapping these impaired reward-related decision-making processes, offering a multi-tiered framework spanning mathematical models, neurochemical markers, behavioral maladaptation, and structural networks. Figure 1 presents a stylized view of the complexity and pathological “short-circuits” occurring within the brain's motivational valuation systems under stress, trauma, or neurodegenerative decay (Figure 1).

**Figure 1:**
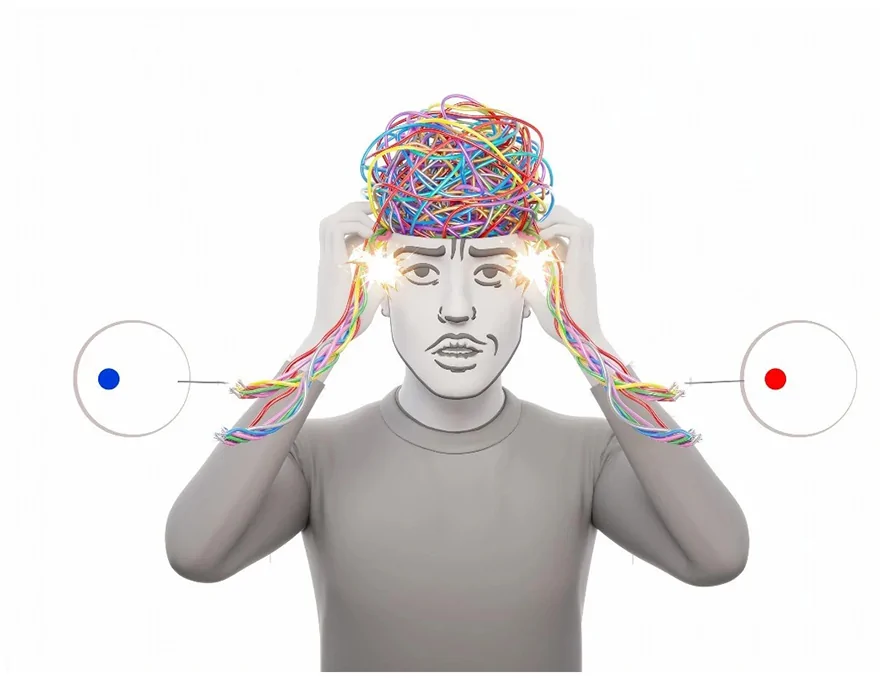
When decision-making networks face a cognitive overload. The stylized figure captures the complexity and pathological “short-circuits” occurring within the brain's motivational valuation systems under stress, trauma, or neurodegenerative decay.

To understand how decision-making goes awry, neuroscientists must first define how the brain evaluates options. The long-standing neuroeconomic hypothesis posits that the brain translates disparate choices—such as food, social status, or money—into a uniform internal metric of subjective value. However, this model has faced intense scrutiny regarding its psychological realism. A very recent meta-analysis has refined this “common currency” view by introducing crucial dimensional separations (Sripada). This meta-analysis demonstrates that common currencies do not operate as a single monolithic signal, but instead function across two distinct phases and at two separate levels:

- The decision-phase vs. the learning-phase: separating the active computation of value during an immediate choice from the subsequent updates made when experiencing a reward or punishment.
- The options-level vs. the meta-level: differentiating the direct evaluation of competing choices from the higher-order control mechanisms that determine how resources are allocated across entire strategies. The paper also highlights a crucial theoretical divergence between cognitive science and economics. Cognitive science relies heavily on mechanistic “common currency models,” which focus on the biological and algorithmic processes that generate value. Conversely, economics relies on axiomatic common currency models tied to expected utility theory, focusing on rational preference structures and behavioral outcomes rather than neural constraints. When psychiatric illnesses strike, they selectively disrupt these localized phases and levels, skewing the delicate balance of value computation.

While algorithmic models track the flow of value, neurochemical imaging reveals the molecular engines behind these math problems. An interesting original research study investigated how neurotransmitter concentrations influence learning under conditions of gains and losses (Park et al.). Using magnetic resonance spectroscopy, researchers measured baseline glutamate/glutamine (Glx) concentrations within the prefrontal cortex and the anterior insular cortex to determine their relationship with general anxiety and depression. The study revealed a crucial neurobiological mechanism: Glx-related error sensitivity within the anterior insular cortex serves as a vital bridge linked to general psychopathology. As individuals attempt to learn from positive and negative feedback, the anterior insula monitors prediction errors and internal visceral states. When local glutamate concentrations are altered, this internal error signaling breaks down, driving the affective miscalculations characteristic of anxiety and depressive disorders. Consequently, this study highlights the anterior insular cortex as a primary target for future glutamate-mediated therapies aimed at recalibrating skewed decision-making processes.

The real-world consequences of value-processing breakdowns are vividly demonstrated in behavioral maladaptation like eating disorders. Looking closely at the intersection of anxiety, depression, and compulsive habits, an innovative original study evaluated how environmental stressors trigger eating pathologies (Vajdová et al.). Utilizing an animal model of compulsive chocolate seeking and taking behavior, researchers exposed subjects to diverse environmental stressors, evaluating both food-related and non-food-related triggers. The findings confirmed that severe stress alters the brain's motivational valuation, driving an aberrant motivation toward palatable foods. When an organism is pushed into a high-stress state, its internal common currency scale tilts violently toward immediate, high-calorie relief, triggering pathological risk-based decision-making. This behavioral shift models the mechanics of binge-eating-like maladaptive behaviors in humans, where the urgent pursuit of reward blinds the decision-making network to long-term physical or psychological consequences.

Pathological choices are not exclusive to functional psychiatric illnesses; they are also prominent features of neurodegenerative decay. A comprehensive review study evaluated the behavioral variant of frontotemporal dementia (bvFTD), a disease characterized by striking changes in personality and social conduct. The review assessed whether impaired decision-making stands as the most prominent and clinically relevant cognitive deficit in these patients, often manifesting early and preceding global cognitive decline (Tavakolan Haghighi et al.). Remarkably, the study revealed that even when bvFTD patients maintain completely intact standard cognitive profiles (such as preserved memory and language skills), they show profound impairments on specialized decision-making tasks. This critical finding suggests that choice deficits may serve as an early, highly specific clinical marker of the disease, long before broader dementia symptoms surface. Neuroimaging data within the review linked these deficits to structural atrophy across a widely distributed network. This degenerative web spans the ventromedial and orbitofrontal cortices, the anterior cingulate, the insula, and even cerebellar regions—the latter increasingly recognized for its role in error-based learning and cognitive tracking.

When decision-making impairments spill into the social domain, they can alter behavior in ways that intersect with the legal system. A key meta-analysis examined the central role of impulsivity in models accounting for criminal conduct in general and violent behavior specifically. A classic metric of impaired choice is delayed reward discounting—the tendency to overvalue immediate rewards while drastically devaluing future consequences (Sebalo et al.). The meta-analysis uncovered an important distinction: while steep delayed reward discounting is robustly associated with general criminal conduct, its specific association with violence is tenuous. Violent acts are often driven by immediate threat-reactivity, social cognitive deficits, or acute neurochemical spikes, rather than the calculated, long-term reward discounting that predicts property or financial crimes. This differentiation warns clinicians and criminologists against treating “impulsivity” as a single monolithic cause of anti-social behavior.

At the furthest end of cognitive disorganization sits schizophrenia, a multiform psychiatric disorder where impairments in high-order cognitive abilities—such as behavioral flexibility, working memory, and decision-making—are considered core onset markers. A visionary review study aimed to synthesize these disparate symptoms into a single, hypothesis-generating framework focused on altered brain communication (Cernousova and Patrono). The study explored the well-known disruption in prefrontal-hippocampal communication, but specifically sought out the intermediate structures contributing to cognitive impairments in psychosis. By gathering translational, *in vivo*, and *in silico* (computational) evidence, the review demonstrated a significant involvement of the midline thalamic nuclei in gating prefrontal-hippocampal traffic. When this midline thalamic hub fails, the brain cannot synchronize its memory systems with its executive planning networks. This failure stimulates a vital need for new computational and translational experimental methodologies, providing a broad, nuanced framework to map maladaptive brain communication and develop targeted interventions for psychosis.

In conclusion, together, this Research Topic of scientific literature demonstrates that pathological decision-making is not a vague behavioral quirk, but a quantifiable neurological failure. Whether mapped via the common currency models of neuroeconomics, tracked through insular glutamate levels, or traced to midline thalamic network disconnections, impaired choice serves as a powerful window into the psychiatric mind. By refining these algorithmic and structural models, modern neuroscience paves the way for targeted, circuit-based therapies that can help patients regain control over their choices and, ultimately, their lives.

